# TSCA Reform Under Way in Congress

**DOI:** 10.1289/ehp.1001917

**Published:** 2010-03

**Authors:** Linda S. Birnbaum

**Affiliations:** National Institutes of Health, Department of Health and Human Services, Research Triangle Park, North Carolina, E-mail: birnbaumls@niehs.nih.gov

Three decades ago, President Gerald Ford signed the federal Toxic Substances Control Act (TSCA 1976) to restrict or ban the use of toxic chemicals. The world has changed remarkably since the inception of TSCA. There are new chemicals and materials today, including those created using nanotechnology—only a dream in 1976. Our understanding of chemical hazards and the techniques used to evaluate potential toxicity have evolved significantly. Now, with this confluence of knowledge and technology, there is broad consensus from a diverse group of federal agencies and other interested parties that the nation is ready to pursue a fresh perspective on chemical evaluation.

To jump-start the discussion on TSCA reform, the U.S. Senate Committee on Environment and Public Works convened a hearing on 2 December 2009. Lisa Jackson, Administrator of the U.S. Environmental Protection Agency (EPA); John Stephenson, Director of the U.S. Government Accountability Office Natural Resources and Environment; and I testified to the Committee chaired by Senator Barbara Boxer of California (U.S. Senate Committee on Environment and Public Works 2009). My peers’ remarks focused on principles for TSCA reform based on industry participation and expansion of the U.S. EPA’s authorities. My primary goal was to inform lawmakers of the progress in environmental health science, which has made tremendous strides due in part to the advancements achieved by the National Institute of Environmental Health Sciences (NIEHS) and the National Toxicology Program (NTP). These advancements, along with critical partnerships between agencies, can set the groundwork for a major shift in evaluating and regulating the safety of tens of thousands of chemicals.

Research has shown that the normal development of the fetus, infant, and child can be disrupted by relatively low doses of certain chemicals. These developmental stages are “windows of susceptibility” when there is increased vulnerability to the effects of toxic chemicals. This concept was first established for neurodevelopmental toxicants such as polychlorinated biphenyls (PCBs), lead, mercury, and other metals, but it also applies to hormonally active agents such as bisphenol A, an endocrine-disrupting chemical. For example, researchers at the Breast Cancer and Environment Research Program (cofunded by the NIEHS and the National Cancer Institute) are determining whether windows of susceptibility exist in the development of the mammary gland, and if exposures to environmental agents during these vulnerable periods of development increase the risk for breast cancer in adulthood. This new understanding heightens the need to protect our children from dangerous substances at critical times in their development.

Toxicity research must extend to health end points beyond those traditionally emphasized—namely, cancer and birth defects. For example, the NIEHS is supporting research on the origins of obesity and the theory that environmental exposures during a child’s development play an important role in the current epidemic of obesity, diabetes, and metabolic syndrome. There are data showing weight gain in rats and mice after developmental exposure to a number of different substances, so we need to start thinking about obesity, not just in terms of genetics and lifestyle, but also in terms of environmental chemicals. These kinds of health outcomes need to be considered in assessing toxicity.

Furthermore, all of us are exposed to many different chemicals at the same time, not just one chemical at a time the way they are usually tested in the laboratory (and currently approved for use). Scientists have successfully estimated risk from combinations of exposures. One example was the method used for dioxin, a known human carcinogen, and related compounds such as furans and some PCBs. A method was developed to estimate toxicity of mixtures of dioxin-like compounds based on toxic equivalency factors (TEFs). The methodology was tested and confirmed by the NTP, the U.S. EPA, and others. The TEF methodology is also applied to other health end points, including reproductive, developmental, immune, and neurologic outcomes. The question for public health officials is how health standards can be adjusted to take into account the fact that people are always exposed to mixtures of compounds, not individual chemicals in isolation.

The route of exposure must also be considered. For example, initial studies on the inhalation of hexavalent chromium showed it causes lung cancer in humans, but there was concern over its presence in drinking water and the safety of ingesting the chemical. Additional studies by the NTP showed that oral consumption of hexavalent chromium causes cancer in laboratory animals, at concentrations that are not that much higher than what can be found in people. Thus, although there is a clear need to test different routes of chemical exposure when assessing toxicity, it is equally important to focus on the internal dose of the chemical in order to compare animals and people.

TSCA reform can be built upon vastly improved and less expensive toxicologic testing methods. The NTP is laying the foundation for this testing paradigm in partnership with the National Human Genome Research Institute and the U.S. EPA. The new methods use quantitative high-throughput screening assays to test a large number of chemicals simultaneously, dramatically increasing the rate at which chemicals can be prioritized for further testing.

Over the past 33 years we have significantly expanded our understanding of chemical exposures and health. We now have the ability to apply new technologies and our growing knowledge to more accurately address the risks of dangerous toxic chemicals. This provides a firm foundation for a comprehensive safety system our citizens deserve under a revitalized TSCA.

## Figures and Tables

**Figure:**
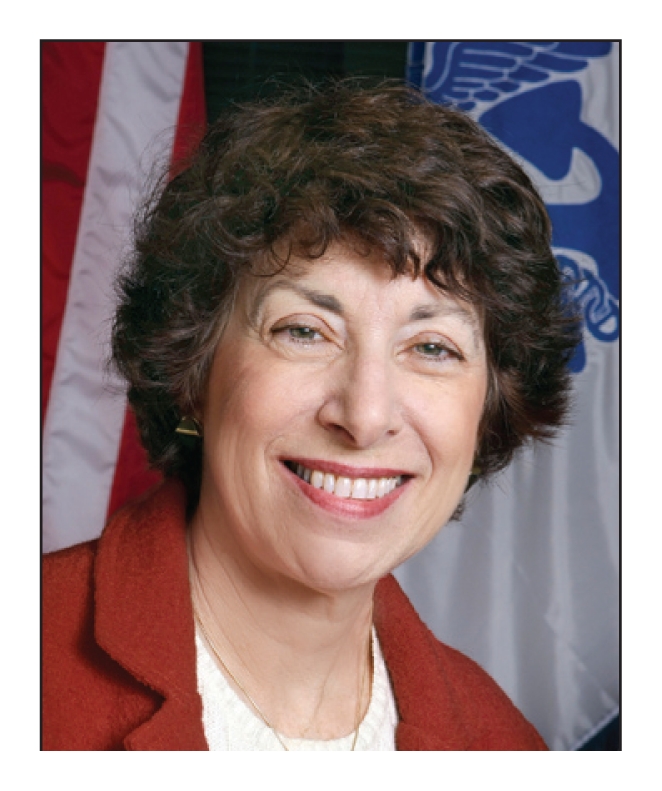
Linda S. Birnbaum
